# PRUNE is crucial for normal brain development and mutated in microcephaly with neurodevelopmental impairment

**DOI:** 10.1093/brain/awx014

**Published:** 2017-02-28

**Authors:** Massimo Zollo, Mustafa Ahmed, Veronica Ferrucci, Vincenzo Salpietro, Fatemeh Asadzadeh, Marianeve Carotenuto, Reza Maroofian, Ahmed Al-Amri, Royana Singh, Iolanda Scognamiglio, Majid Mojarrad, Luca Musella, Angela Duilio, Angela Di Somma, Ender Karaca, Anna Rajab, Aisha Al-Khayat, Tribhuvan Mohan Mohapatra, Atieh Eslahi, Farah Ashrafzadeh, Lettie E. Rawlins, Rajniti Prasad, Rashmi Gupta, Preeti Kumari, Mona Srivastava, Flora Cozzolino, Sunil Kumar Rai, Maria Monti, Gaurav V. Harlalka, Michael A. Simpson, Philip Rich, Fatema Al-Salmi, Michael A. Patton, Barry A. Chioza, Stephanie Efthymiou, Francesca Granata, Gabriella Di Rosa, Sarah Wiethoff, Eugenia Borgione, Carmela Scuderi, Kshitij Mankad, Michael G. Hanna, Piero Pucci, Henry Houlden, James R. Lupski, Andrew H. Crosby, Emma L. Baple

**Affiliations:** 1 Dipartimento di Medicina Molecolare e Biotecnologie Mediche DMMBM, Università di Napoli Federico II, Via Sergio Pansini 5, Naples, 80131, Italy; 2 CEINGE Biotecnologie Avanzate, Via Gaetano Salvatore 486, Naples, Italy; 3 European School of Molecular Medicine, SEMM, University of Milan, Italy; 4 Medical Research (Level 4), RILD Wellcome Wolfson Centre, University of Exeter Medical School, Royal Devon & Exeter NHS Foundation Trust, Barrack Road, Exeter, EX2 5DW, UK; 5 Department of Molecular Neuroscience, UCL Institute of Neurology, London, UK; 6 Section of Ophthalmology and Neuroscience, Leeds Institute of Biomedical and Clinical Sciences, University of Leeds, UK; 7 National Genetic Centre, Directorate General of Royal Hospital, Ministry of Health, Muscat, Sultanate of Oman; 8 Molecular Genetics, Department of Anatomy, Institute of Medical Sciences, Banaras Hindu University, Varanasi -221005, UP, India; 9 Department of Medical Genetics, School of Medicine, Mashhad University of Medical Sciences, Mashhad, Iran; 10 Medical Genetics Research Center, School of Medicine, Mashhad University of Medical Sciences, Mashhad, Iran; 11 Dipartimento di Scienze Chimiche, Università Federico II, Naples, Italy; 12 Department of Molecular and Human Genetics, Baylor College of Medicine, Houston, TX 77030, USA; 13 Department of Biology, Sultan Qaboos University, PO Box 36, Post code 123, Sultanate of Oman; 14 Department of Pediatric Neurology, Ghaem Medical Center, School of Medicine, Mashhad University of Medical Sciences, Mashhad, Zip Code– 9919991766, Iran; 15 Department of Pediatrics, Institute of Medical Sciences, Banaras Hindu University, Varanasi –221005, UP, India; 16 Department of Psychiatry, Institute of Medical Sciences, Banaras Hindu University, Varanasi –221005, UP, India; 17 Department of Medical and Molecular Genetics, Division of Genetics and Molecular Medicine, King's College London, London, UK; 18 Department of Neuroradiology, St. George's Hospital, London, UK; 19 Genetics Research Centre, St. George's, University of London, London, SW17 0RE, UK; 20 Unit of Neuroradiology, Department of Biomedical Science and Morphological and Functional Images, University of Messina, Messina, Italy; 21 Unit of Child Neurology and Psychiatry, Department of Human Pathology of the Adult and Developmental Age, University of Messina, Messina, Italy; 22 Unit of Neuromuscular disorders, IRCCS Oasi Maria SS Troina, Enna, Italy; 23 Department of Neuroradiology, Great Ormond Street Hospital for Children, London WC1N 3JH, UK; 24 MRC Centre for Neuromuscular Diseases, UCL Institute of Neurology, London WC1N 3BG, UK; 25 Human Genome Sequencing Center, Baylor College of Medicine, Houston, TX 77030, USA; 26 Department of Pediatrics, Baylor College of Medicine, Houston, TX 77030, USA; 27 Texas Children's Hospital, Houston, TX 77030, USA

**Keywords:** *PRUNE1*, microcephaly, developmental delay, normal brain development, microtubule polymerization, tubulinopathy

## Abstract

PRUNE is a member of the DHH (Asp-His-His) phosphoesterase protein superfamily of molecules important for cell motility, and implicated in cancer progression. Here we investigated multiple families from Oman, India, Iran and Italy with individuals affected by a new autosomal recessive neurodevelopmental and degenerative disorder in which the cardinal features include primary microcephaly and profound global developmental delay. Our genetic studies identified biallelic mutations of *PRUNE1* as responsible. Our functional assays of disease-associated variant alleles revealed impaired microtubule polymerization, as well as cell migration and proliferation properties, of mutant PRUNE. Additionally, our studies also highlight a potential new role for PRUNE during microtubule polymerization, which is essential for the cytoskeletal rearrangements that occur during cellular division and proliferation. Together these studies define PRUNE as a molecule fundamental for normal human cortical development and define cellular and clinical consequences associated with PRUNE mutation.

## Teaser

Zollo *et al*. report that mutations in *PRUNE1*, a phosphoesterase superfamily molecule, underlie primary microcephaly and profound global developmental delay in four unrelated families from Oman, India, Iran and Italy. The study highlights a potential role for prune during microtubule polymerization, suggesting that prune syndrome may be a tubulinopathy.

## Introduction

PRUNE first identified in *Drosophila* through impairing the formation of eye pigments (drosopterins), belongs to the DHH family of phosphoesterases (Timmons *et al.*, 1995). Subsequent studies indicated that PRUNE cAMP-phosphodiesterase activity is important for cell motility (D’Angelo *et al.*, 2004) with a prominent exopolyphosphatase activity, and that PRUNE-interacts with glycogen synthase kinase-3 (GSK-3β), a negative regulator of canonical WNT/β-catenin signalling (Diana *et al.*, 2013; Carotenuto *et al.*, 2014). Consistent with this role PRUNE overexpression has also been shown to correlate with the staging of colorectal cancer liver metastases (Hashimoto *et al.*, 2016), and PRUNE expression is an independent predictor of survival of patients with gastric cancer (Oue *et al.*, 2007). While a clear molecular role for PRUNE in brain development has not been previously demonstrated, PRUNE’s binding partner GSK-3β is a crucial inhibitory regulator of many neuronal functions including neurite outgrowth, synapse formation, neurogenesis and survival, which may be mediated via GSK-3β promotion of apoptotic signalling in cultured neural precursor cells (Eom *et al.*, 2007). In addition, neuronal overexpression of GSK-3β has been shown to result in delayed postnatal maturation and differentiation of neurons in the mouse brain (Spittaels *et al.*, 2000, 2002). Further evidence for a role of PRUNE in neurodevelopment is provided by studies of the temporo-spatial expression pattern of Prune during mouse brain development, which revealed strong expression in multiple brain regions during early development indicative of a role in neurogenesis and neuronal migration (Carotenuto *et al.*, 2006). Recently, Karaca and colleagues identified biallelic mutations in *PRUNE1* as a candidate genetic cause of microcephaly, cortical atrophy, thin or hypoplastic corpus callosum, cerebellar atrophy and global developmental delay in five affected individuals (Karaca *et al.*, 2015). In the current study we confirm a key role for PRUNE in human brain development by defining PRUNE mutations in 13 individuals in extended families from Oman and Iran, as well as two smaller families from India and Italy, affected by overlapping clinical features. Together with our functional assessments of these PRUNE mutations, our data enable a more detailed clinical comparison to be drawn between the patient cohort described here with the five patients in whom candidate PRUNE mutations were recently defined (Karaca *et al.*, 2015), permitting us to more precisely define the molecular basis and clinical outcome arising from PRUNE mutation.

## Materials and methods

### Patient ascertainment and genetic studies

The present studies were reviewed and approved by the local authorities in the Sultanate of Oman, Iran, India and Italy and all tissue samples were taken with informed consent and in accordance with the local government guidelines on research governance. DNA and RNA were extracted from blood or buccal samples using standard techniques. Single nucleotide polymorphism (SNP) genotyping was carried out using Illumina Human CytoSNP-12v2.1 arrays. Multipoint linkage analysis was performed with Simwalk (Sobel *et al.*, 2001) under a model of autosomal-recessive inheritance with full penetrance. Unique primers for PCR and sequencing were designed using online software Primer3web (Koressaar and Remm, 2007; Untergasser *et al.*, 2012) using sequences from the UCSC Genome Browser. Bidirectional dideoxy DNA sequencing was performed on an ABI3130 XLA capillary sequencer (Applied Biosystems) with analysis using Finch TV 1.4.0 (Geospiza Inc.) and Gene Tool 1.0.0.1 (Bio Tools Inc.). Whole-exome sequence analysis (Family 1) was performed by in-solution hybridization (Sure Select system, Agilent) on Illumina GAIIx. Sequence reads were aligned with the Novoalign software package (Novocraft Technologies Sdn Bhd) and duplicate reads excluded from downstream analysis. Depth and breadth of sequence coverage was calculated with custom scripts and the BedTools package (Quinlan and Hall, 2010). Single nucleotide substitutions and small insertion deletions were identified and quality filtered within the SamTools software package (Li *et al.*, 2009) and in-house software tools. Variants were annotated with respect to genes and transcripts with the Annovar tool (Wang *et al.*, 2010).

### Immunoprecipitation of FLAG-PRUNE complexes

Complexes were purified by immunoprecipitation from total extracts from breast carcinoma cells as described (Carotenuto *et al.*, 2006). Total extracts were pre-cleaned on mouse IgG agarose beads (Sigma) for 2 h at 4°C to reduce non-specific binding and then incubated with M2 anti-FLAG agarose-conjugated antibody (Sigma) for 4 h at 4°C. Elution of immunoprecipitate was performed with 3× FLAG peptide in buffer A. The eluted extracts were precipitated with methanol/chloroform before loading on 10% polyacrylamide SDS-PAGE. The gel was stained with Coomassie colloidal blue (Pierce). Protein bands were excised from the gel, reduced and carboxy-amidomethylated with 10 mM DII and 55 mM iodoacetamide in 50 mM NH_4_HCO_3_ buffer pH 8 and *in situ* digested with trypsin.

### Cell culture

Cells were cultured in DMEM (HEK293 and HELA) or RPMI (SHSY5Y) medium with 10% foetal bovine serum (FBS), 2 mM l-glutamine, and 1% penicillin/streptomycin. SHSY5Y cells were transfected with the pcDNATM6/IR plasmid (encoding the Iet repressor, IetR) using Lipofectamine^®^ 2000 (Invitrogen). Cellular clones were generated by selection with blasticidin (5 µg/ml) for 10 days. Positive clones were then transfected with the episomal pT-REx-DEST30 vectors encoding PRUNE wild-type, PRUNE-D30N, PRUNE-R297W or empty vector as a control. Cellular clones were generated by selection with Neomycin (500 µg/ml). Ir6/ pT-REx-DEST30 PRUNE-positive cells were determined by western blot analysis 24 h after the addition of doxycycline at a final concentration of 2 µg/ml.

### Cell index assay

Cell migration and proliferation were assessed using Roche xCELLigence system. HEK293 cells transfected with wild-type, D30N and R297W PRUNE expressing plasmids were harvested and washed with phosphate-buffered saline (PBS) and then resuspended in Dulbecco’s modified Eagle medium (DMEM) with 0% FBS. Each cell suspension was then added to a single well on the xCELLigence CIM-plate 16. Migration was driven by a 10% FBS gradient (as oval coloured) and 0% FBS gradient as control (as circle coloured), and assessed at 5-min intervals by measuring impedance changes across electrodes at the bottom of the wells. Cellular proliferation assays were performed by adding SH-SY5Y inducible clones (5 × 10^3^) to single wells of the xCELLigence E-plate 16. Adenovirus particles AdV-UNR (unrelated) and AdV-sh-PRUNE expressing sequences silencing endogenous PRUNE translation were added to the cells and then expression of wild-type, R297W and D30N mutated PRUNE proteins expression was induced adding doxycycline after 2 h of virus infection. Proliferation rates were determined by measuring electrical impedance changes in the electrodes at the bottom of each well at 2-min intervals for 26 h (proliferation assay) and 12 h (migration assay).

### Microtubule nucleation assays and immunofluorescence analyses

SH-SY5Y inducible clones were plated and grown for 24 h in the presence of doxycycline. Then, inducible cells growing on the coverslips were incubated on ice for 1 h and then at 30°C for 2 min. Cells were fixed in 4% paraformaldehyde and permeabilized for 10 min in phosphate buffer containing 0.1% Triton^™^ X-100, before incubation with 0.1% Triton^™^ X-100, 10% pig serum and either anti-β-Tubulin (1 µg/ml; Abcam, ab15568), anti-PRUNE (1 µg/ml; Abcam, ab88613) antibodies. Confocal microscopy was carried out using a laser scanning confocal microscope LSM 510 META, Zeiss, with 40×/63× water immersion objectives.

### *In vitro* microtubule polymerization assay

Prune ability to promote microtubule assembly was determined using the Tubulin polymerization assay kit according to the manufacturer’s instructions (Cytoskeleton, #BK011P). Briefly, the standard polymerization reaction containing 50 μl volume of 2 mg/ml tubulin in 80 mM PIPES pH 6.9, 0.5 mM EGTA, 2 mM MgCl_2_, 1 mM GTP, 10% glycerol. Polymerization was started by incubation at 37°C and followed by absorbance readings at 360 nm. The standard polymerization reactions were performed alone and in the presence of 3 μM paclitaxel, 3 μM nocodazole or of 800 ng wild-type, D30N or R297W purified proteins. EnSpire software manager v. 4.13.3005.1482 was then used to calculate the kinetic slope of the independent assays.

### Differentiation assay

SH-SY5Y inducible clones were treated with doxycycline (DOX) to induce expression of wild-type, R297W and D30N mutated PRUNE proteins, and with all-trans retinoic acid (ATRA, 20 µM) to induce neuronal differentiation, untreated clones were included as a control. Culture medium was changed and differentiation assessed at Day 7 by real-time polymerase chain reaction (PCR) measurement of levels of TUJ1 expression as a marker of neuronal precursors differentiation.

### Real time PCR and western blotting

Total cellular RNA was extracted using TRIzol^®^ (Invitrogen) according to the manufacturer’s instructions. RT-PCR was carried out using total RNA (1 μg) and the iScript^TM^ cDNA Synthesis kits (Bio-Rad). Transcripts were amplified by real-time quantitative PCR using a 7900 Real-Time PCR System (Applied Biosystems) with Power SYBR^®^ green Master Mix (Bio-Rad). Ct values were normalized to β-actin. Relative expression of *TUBB3* (Tuj1) was determined by the 2^–ΔΔCt^ method. Data are presented as mean ± standard error of two replicate experiments. Absence of endogenous expression of PRUNE in HEK293 cells and reduction of levels of expression of PRUNE after adenovirus infection of SHSY5Y cells was confirmed by western blotting as described previously (Carotenuto *et al.*, 2014). Immunoblotting for the immunoprepicipitation and co-sedimentation assays were performed using the following antibodies: anti-β-Tubulin (1 µg/ml; Abcam, ab15568), anti-alpha Tubulin (1 µg/ml; Abcam, ab15246), anti-Flag (1 µg/ml; Sigma, F3165), anti-Kinesin (1 µg/ml; Abcam, ab62105) and anti-GAPDH (1 µg/ml; Abcam, ab37168).

### Cell-based microtubules-binding proteins spin-down assay

Cell-based co-sedimentation assay was performed as previously described (Darshan *et al.*, 2011; Sung and Giannakakou, 2014; Friese *et al.*, 2016). Briefly, SHSY5Y inducible cell clones expressing Flag-tagged wild-type PRUNE or empty vector were treated with doxycycline for 24 h. The cells were washed in cold PBS and lysed in cell lysis buffer (50 mM TRIS-HCl pH 7.4, 5 mM MgCl_2,_ 0.1 mM EGTA, 0.5% Triton^™^ X-100 and cocktail protease inhibitors from Roche). Lysates were cleared by centrifugation at 16 200*g* for 15 min at 4°C, and the supernatants were removed and assayed for protein concentration using the Protein Assay Dye Reagent (Bio-Rad). Twenty microlitres of porcine brain tubulin (10 mg/ml; #T240-DX; Cytoskeleton) was supplemented with 1 mM GTP, 1 mM EGTA, 1 mM DTT and 80 μl of BRB80 buffer (#BP01; Cytoskeleton) consisting of 80 mM PIPES, 2 mM MgCl_2_, 0.5 mM EGTA, pH 6.9 and allow to polymerize at 37°C for 30 min by adding paclitaxel (#TXD01; Cytoskeleton) drop wise (starting from 0.02 mM up to 2 mM). Microtubules were kept at room temperature. Fifty microlitres of polymerized microtubules were incubated with fifty micrograms of whole protein extracts from SHSY5Y cells expressing Flag-tagged wild-type PRUNE or empty vector for 30 min at 30°C. Samples were loaded into cushion buffer (1 M sucrose in 50 mM TRIS-HCl, pH 6.9) and microtubules were pelleted by centrifugation at 100 000 *g* at room temperature in an ultracentrifuge (Beckman Coulter, Inc.). The supernatant (S) was separated and the pellet (P) was resuspended in an equal volume of RIPA buffer consisting of [20 mM sodium phosphate, pH 7.4, 150 mM NaCl, 10% (v/v) glycerol, 1% (w/v) Na-deoxycholate, and 1% (v/v) Triton^™^ X-100] supplemented with protease inhibitors (Roche). Protein concentration from both supernatant and pellet was estimated with Bradford reagent (Bio-Rad protein assay). Twenty micrograms from each fraction (P and S) were loaded in a 10% SDS-PAGE and immunoblotting was done with antibodies anti-Flag (1 µg/ml; Sigma, F3165), anti-Kinesin (1 µg/ml; Abcam, ab62105) and anti-β-Tubulin (1 µg/ml; Abcam, ab15568).

### Cloning of *PRUNE1* and variants for *Escherichia coli* protein expression

Wild-type *PRUNE1* construct (amino acid residues 1–393) was cloned using TOPO technology into pET151/D-TOPO (Invitrogen), resulting in a construct with a His-tag fused to the N-terminus of PRUNE via a linker consisting of a TEV (tobacco etch virus) protease cleavage site. The mutations (D30N and R297W) were introduced via site-directed mutagenesis using mutagenic primers designed with a mismatch in the centre of the oligonucleotide, and sequencing verified. His-tagged wild-type and variant *PRUNE1* pET151 constructs were then transformed into *E. coli* Rosetta 2 strain.

### Expression and purification of PRUNE and mutant proteins from *Escherichia coli*

*E. coli* transformed cells were grown in LB medium at 37°C with 50 µg/ml ampicillin to an optical density of ∼0.6 OD at 600 nm when 1 mM isopropyl-beta-d-thiogalactopyranoside (IPTG) was added. The culture was grown over night at 22°C for PRUNE production. Cells were harvested by centrifugation at 1000 rpm for 20 min at 4°C. The cellular pellet was resuspended in equilibration buffer (20 mM Na_2_HPO_4_, pH 7.4, 500 mM NaCl, 20 mM imidazole) containing 1 mM PMSF and disrupted by French Press. The cell extract was centrifuged at 13 000 rpm for 20 min at 4°C and the supernatant was filtered with a syringe-driven filter (0.22 µm) before protein purification. Soluble cell extract was loaded onto a HIS-Select® Nickel Affinity Gel equilibrated with equilibration buffer and the bound protein was eluted by a 12 ml linear gradient from 20 to 500 mM imidazole. Protein concentration was estimated with Bradford reagent (Bio-Rad protein assay), protein purity was assessed by SDS-PAGE.

### Enzymatic activity of PRUNE

The activity of PRUNE on P4 substrate was determined with a fixed-time assay using BIOMOL® Green phosphate reagent (Biomol). The reaction was performed for 60 s in 50 µl of 0.1 M Tris-HCl pH 7.2, containing 50 µM EGTA, 10 mM Mg ions at a substrate concentration ranging from 0 µM to 250 µM on a microtitre plate. The reaction was stopped by addition of 100 µl BIOMOL® Green reagent and the increase in absorbance at 620 nm was measured following 25 min incubation. Kinetic parameters were fitted by non-linear regression with GraphPad Prism 4Project.

### Statistical analyses

All of the data are presented as mean ± standard error. Statistical significance was calculated using the Mann-Whitney U-test. The difference was considered statistically significant at *P* < 0.05. All statistical analyses were performed using the SPSS 16 statistical package for Windows.

## Results

### Prune syndrome phenotype

Supplementary Table 1 summarizes the core phenotypic features of 13 individuals, not previously reported, aged between 3 months and 21 years. Additional clinical details are provided on two Turkish patients (Patients BAB3500 and BAB3737) previously described (Karaca *et al.*, 2015)*.*Supplementary Fig. 1 illustrates the clinical features and MRI scans of affected individuals from each family.

Reduced foetal movements compared to healthy siblings was a prenatal sign reported by some mothers of Prune syndrome children. Microcephaly was progressive and psychomotor development was severely delayed in all domains. Affected individuals did not communicate, and none achieved purposeful hand movements or independent ambulation. Central hypotonia and in some cases bilateral talipes were present at birth. Peripheral spasticity, clonus, muscle wasting and multiple joint contractures developed with advancing age. Slowing of the peripheral nerve conduction velocity at the neurophysiological investigations performed at 2 years of age was seen in the Italian boy. A history of generalized tonic clonic seizures from early infancy was reported in four of the affected children. The oldest of the two Italian children developed infantile spasms at 4 months of age and the younger myoclonic seizures at a similar age. Neuroimaging revealed focal white matter changes, delayed myelination, cortical atrophy, thin or hypoplastic corpus callosum, and cerebellar atrophy. Serial MRI scans in the Italian boy showed severe progression of the disease with increasing age, with delayed myelination reported at 6 months of life and follow-up MRI at the age of 18 months showing diffuse abnormalities of the white matter, progressive brain atrophy involving the cerebral cortex and most strikingly the cerebellum. Craniofacial features included a sloping forehead and large prominent ears and eyes consistent with microcephaly. The Italian girl was reported to have intermittent facial swelling and pedal oedema. Plagiocephaly, a narrow palate, scoliosis and a narrow chest were frequently observed across the studied families and most likely attributable to the severe central hypotonia. The two Italian children were both found to have optic atrophy, documented in both by the age of 2 years. Congenital cataract was an additional feature seen in one of the Iranian children and both of the affected Indian siblings. More detailed ocular phenotyping in a larger series of Prune syndrome patients is required to evaluate if these are real associations with the condition or coincidental and the result of a second autosomal recessive condition in these families. The cause of death in the affected children was respiratory failure, presumed secondary to aspiration.

### Genetic studies

**Figure 1:**
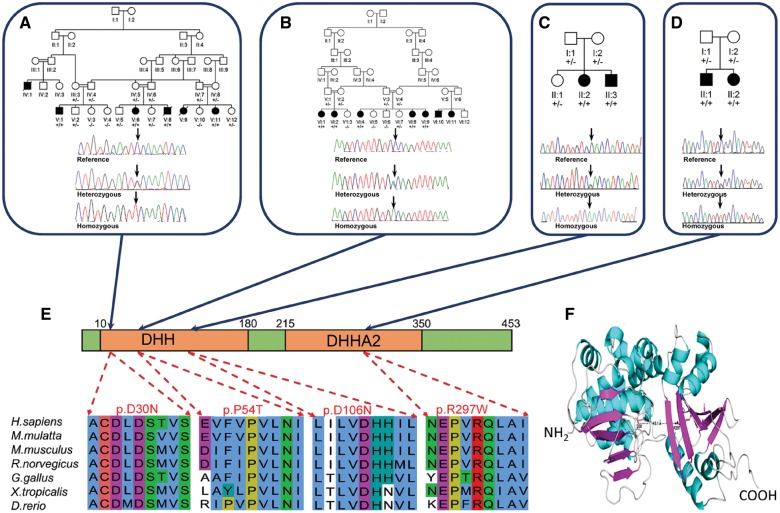
**Family pedigrees, genotype and PRUNE mutation.** Mutations in *PRUNE1* detected in Omani (**A**); Iranian (**B**); Italian (**C**) and Indian (**D**) families. (**E**) Alignment of PRUNE amino acid sequence showing stringent conservation of the Asp30; Pro54; Asp106 and Arg297 residues. (**F**) 3D model of PRUNE showing the location and close proximity of the Asp30 and Arg297 amino acid residues.

To map the chromosomal location of the causative gene, we performed high density genome-wide SNP mapping assuming that a founder mutation was responsible for the disease in Families A, B and D. This identified a single notable homozygous region of 10.1 Mb of chromosome 1q21.3 in Family A demarcated by rs12033302 and rs11264516, considered likely to correspond to the disease locus. Multipoint linkage analysis was performed on Family A (Sobel *et al.*, 2001), under a model of autosomal-recessive inheritance with full penetrance, producing a highly significant logarithm of the odds (LOD) score across chromosome 1q21.3 (LOD_max_ = 6.07). This region overlapped one of only two notable regions of homozygosity identified in Family B (102.1 Mb demarcated by rs3855975 and rs2274316), as well as one of the two small regions of homozygosity identified in Family D, which spans 3.7 Mb (demarcated by SNPs rs7513205 and rs11265303; Fig. 1 and Supplementary Fig. 2). To identify the causative mutation, we performed whole exome sequencing of a single affected individual from each of Families A and B. After filtering the identified variants for call quality, potential pathogenicity, population frequency and localization within the candidate interval, we identified only a single likely deleterious variant in Family A, a c.88G > A (p.Asp30Asn) alteration in *PRUNE1* (NM_021222.2), as the primary candidate mutation. Additionally, exome sequencing identified a c.160C > A (p.Pro54Thr) *PRUNE1* sequence variant in Family B and a c.316G > A (p.Asp106Asn) in Family C. A c.889C > T (p.Arg297Trp) alteration in *PRUNE1* was subsequently identified in Family D following dideoxy sequencing of all coding exons of this gene (Fig. 1A–D).

Each variant affects a stringently conserved amino acid residue (Asp30, Pro54, Asp106 and Arg297) located within the two motifs DHH and DHHA2, which are adjacently located inside the predicted enzymatic pocket (Fig. 1E and F). Each mutation was found to co-segregate in each appropriate family consistent with the mode of inheritance, and showed high damage prediction using *in silico* prediction programs [PROVEAN (Choi *et al.*, 2012), PolyPhen-2 (Majava *et al.*, 2007), and SIFT (Ng and Henikoff, 2001)]. The c.88G > A (p.Asp30Asn) and c.160C > A (p.Pro54Thr) alterations were absent from online genomic databases [1000 Genomes (The Genomes Project, 2015), National Heart, Lung, and Blood Institute Exome Sequencing Project database ESP6500SI-V2 release, Exome Aggregation Consortium (ExAC) (Lek *et al.*, 2016)]. The c.316A > G variant was identified in only five heterozygotes in the ExAC database and the c.889C > T (p.Arg297Trp) alteration was reported as a single somatic nucleotide variant (COSM462922) and one heterozygote (South Asian) is listed on the ExAC database. Additionally, the c.88G > A (p.Asp30Asn) mutation of Family A and the c.316G > A (p.Asp106Asn) mutation of Family C have been both previously identified by Karaca *et al.*, (2015) in affected consanguineous families from Saudi Arabia and Turkey, respectively.

### PRUNE mutation impairs cell differentiation, proliferation and migration properties

**Figure 2:**
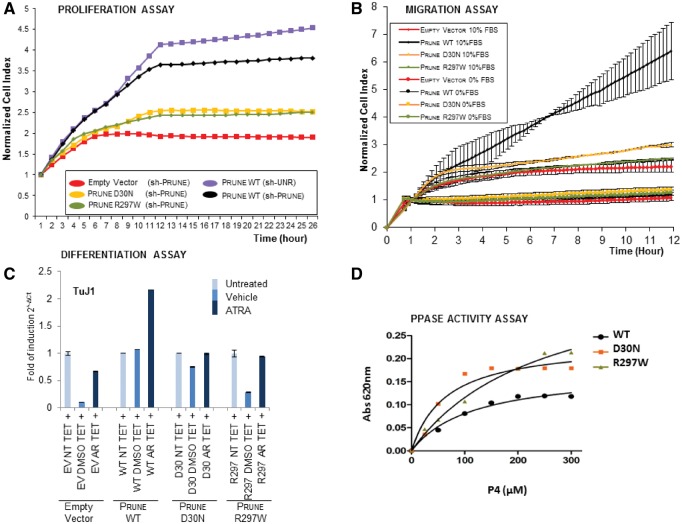
**Impact of PRUNE mutations on known PRUNE functions.** (**A**) Graphs showing normalized cell index as a measure of proliferation of AdV-sh-Prune treated, PRUNE FLAG, PRUNE D30N-FLAG and PRUNE R297W-FLAG cells, stimulated with doxycycline (DOX). Proliferation of Ad-sh-UNR treated PRUNE-FLAG cells was followed as control (light blue circle). Cell proliferation is shown as cell index after normalization to the last cell index recorded before the addition of doxycycline. Data are expressed as mean ± SD of samples assayed in triplicate. (**B**) Graphs showing cell index as a measure of migration of HEK293 cells transfected with plasmids encoding wild-type D30N or R297W PRUNE generated by xCELLigence RICA. Migration kinetics, shown as cell index, were monitored in response to 10% FBS (oval colours) and to 0% FBS (circle colours) as negative control. Data are expressed as mean ± SD of samples assayed in triplicate. (**C**) mRNA expression levels of TUBB3 (TuJ1) in SH-SY5Y PRUNE-wild-type, PRUNE-D30N and PRUNE-R297W cells treated with doxycycline and all-trans retinoic acid (ATRA) for 7 days as determined by RT-PCR. The levels of mRNA expression are represented as fold-multiples of 2−dCt values relative to untreated expression. Data are means (mRNA expression 2−dCt) ± SD (*n* = 3) (^**^P < 0.005). EV = empty vector; NT = not treated; TET = tetracycline (or DOX); AR = all Trans retinoic acid. (**D**) The biochemical activity of both wild-type and mutated (D30N and R297W) PRUNE on tetraphosphates (P4) substrate was determined with a fixed-time assay using BIOMOL^®^ Green phosphate reagent. The increase in the absorbance at 620 nm was measured. Kinetic parameters were fitted by non-linear regression with GraphPad Prism 4Project. Both D30N (orange curve) and R297W (green curve) PRUNE proteins show a higher biochemical activity compared to that of wild-type PRUNE (black curve). WT = wild-type.

*PRUNE1* encodes a 453 amino acid protein that is highly conserved across many species and shares functional properties with the phosphoesterases and the exopolyphosphatase family of proteins due to the presence of the DHH motif (D’Angelo *et al.*, 2004; Tammenkoski *et al.*, 2008). To determine the molecular mechanism by which PRUNE may regulate neurogenesis, we investigated the outcome of PRUNE mutation on its’ previously documented functional roles in cell migration, proliferation and differentiation (D’Angelo *et al.*, 2004; Carotenuto *et al.*, 2006). We investigated two of the mutations identified located in distinct regions of PRUNE, affecting amino acid residues located in separate functional domains so as to define and compare functional outcomes on molecular function; p.D30N identified in this study and the study by Karaca *et al.* (2015) (located in the DHH motif), as well as p.R297W identified in this study (located in the DHHA2 domain). As has been shown previously, our studies here determined that PRUNE silencing profoundly decreased cell proliferation (Fig. 2A and Supplementary Fig. 3D). However, while treatment with wild-type PRUNE returned cellular proliferation rates to normal, treatment with either p.D30N or p.R297W mutant proteins did not (Fig. 2A). In parallel with this we also assessed another known property of PRUNE in enhancing cell migration using HEK293 cells, which display negligible levels of endogenous PRUNE expression (Carotenuto *et al.*, 2014), with expression levels assessed by western blotting (Supplementary Fig. 3E). Unlike wild-type PRUNE, which as expected was found to substantially promote cellular migration, both p.D30N and p.R297W mutants displayed negligible migration promoting activity and were comparable to empty vector controls (Fig. 2B). Next, we also examined the effect of both mutations on cell differentiation in SH-SY5Y cells by measuring the expression level of TuJ1 after treatment with retinoic acid. While wild-type PRUNE promoted a 2-fold increase in neuronal cell differentiation levels, no cellular differentiation was promoted by the PRUNE mutants (Fig. 2C). Finally, the exopolyphosphatase activity assay (Tammenkoski *et al.*, 2008) measuring Kcat/Km ratios versus P4-tetraphosphates substrates, show that both mutant PRUNE (p.D30N and p.R297W) proteins retain a higher exopolyphosphatase activity compared to the wild-type protein (Kcat/Km values wild-type: 0.014 μM/s; D30N: 0.312 μM/s; R297W: 0 064 μM s^−1^; Fig. 2D and Supplementary Table 2).

### PRUNE interacts with β-tubulin, and PRUNE mutation impairs microtubule polymerization activity

**Figure 3:**
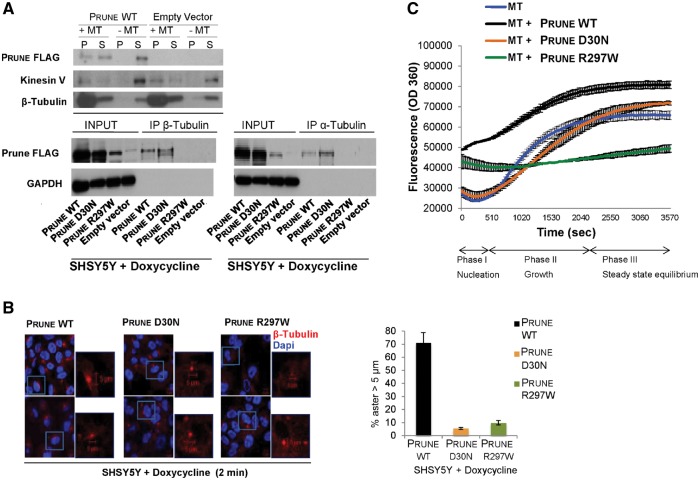
**PRUNE and tubulin.** (**A**) *Top*: Cell-based microtubules co-sedimentation assay and SDS/PAGE analysis showing the binding of FLAG-tagged PRUNE to microtubule polymers (MT) using *in vitro* whole protein extracts from SHSY5Y clones overexpressing wild-type FLAG-tagged PRUNE or empty vector (as negative control), with immunoblotting with antibodies against anti-FLAG, anti-β-tubulin and anti-kinesin V (as a positive control due to its known binding to microtubule polymers). Wild-type FLAG-tagged PRUNE was found in the pellet (P) fraction in the presence of microtubule polymers, while it was found only in the supernatant (S) fraction in the absence of microtubule polymers, indicating microtubule binding. *Bottom*: Co-immunoprecipitation assay using Flag-tagged wild-type, D30N and R297W PRUNE protein expression in SHSY5Y inducible cell clones. The whole protein extract from empty vector (EV, as negative control), wild-type, D30N and R297W PRUNE-overexpressing cells incubated with antibodies against β-tubulin or α-tubulin to immunoprecipitate (IP), endogenous β-tubulin (*left*) or α-tubulin (*right*). A band of the expected size (60 kDa) was detected by western blotting using an anti-Flag antibody in the immunoprecipitate fraction from wild-type and D30N-overexpressing clones, indicating binding of PRUNE wild-type and D30N with both β- and α-tubulin. Flag-tagged R297W PRUNE was detected with a long exposure (Supplementary Fig. 2A and 2B). (**B**) Microtubule nucleation assay. SHSY5Y-inducible cells overexpressing wild-type, D30N, R297W PRUNE proteins were treated with doxycycline followed by immunofluorescence staining with β-tubulin antibody (red), and DAPI for DNA staining (blue). Cells containing microtubule asters with a diameter longer than 5 µm were scored and the results from a representative experiment in triplicate are shown. *Left*: The immunofluorescence analysis performed on the inducible clones after 2 min at 37°C showing some representative asters (in red) for each clone (Scale bars = 5 µm). The chart on the *right* indicates the percentage of cells with aster diameters longer than 5 µm. Wild-type PRUNE expressing clones show a higher percentage of cells containing asters longer than 5 µm, compared to those expressing D30N and R297W PRUNE (∼160 nuclei per clone were counted; Supplementary Table 3). (**C**) *In vitro* microtubule polymerization assay performed using wild-type (black), D30N (orange) and R297W (green) PRUNE purified from *E. coli.* The standard polymerization reaction, alone or in presence of the purified wild-type or mutated (D30N and R297W) PRUNE protein, incubated with tubulin and followed by absorbance readings at 360 nm (excitation at 360 nm, and emission at 420 nm; EnSpire manager software) to evaluate the maximum absolute curve slope. Polymerization curves are shown for the three phases of polymerization; I (nucleation), II (growth), III (steady state). The polymerization rate is enhanced (∼2-fold) in presence of wild-type PRUNE (black) in comparison with microtubules alone (blue). Polymerization in the presence of D30N PRUNE (orange) is unaffected, while it is unregulated by R297W PRUNE (green). Both mutations result in a notable delay of microtubule polymerization rate, which is particularly evident during the nucleation phase (phase I). The curves shown represent the average of *n* = 3 independent experiments, expressed as mean ± SD of samples assayed in triplicate. See Supplementary Fig. 3F for standard polymerization alone, and in the presence of 3 µM paclitaxel or 3 µM nocodazole, used as positive and negative controls, respectively.

To gain further insight into the molecular mechanism by which PRUNE may regulate neurogenesis, we performed a mass-spectrometry-based interaction screen of proteins co-immunoprecipitated by wild-type PRUNE. This identified a number of different proteins involved in cytoskeleton organization as likely binding partners (data not shown). Among these, a putative interaction between PRUNE and tubulin, in particular β-tubulin, was most notable given previous studies highlighting the critical role of tubulins and microtubule-associated proteins during brain development, which may be mutated in autosomal recessive primary microcephaly (MCPH) (Woods *et al.*, 2005; Bahi-Buisson *et al.*, 2014; Sun and Hevner, 2014). We performed a cell-based microtubule-binding proteins spin-down assay (Darshan *et al.*, 2011) with genetically modified SHSY5Y neuroblastoma cells to result in inducible clones overexpressing PRUNE wild-type or mutants, under a tetracycline inducible promoter (see ‘Material and methods’ section and Supplementary Fig. 3C). Upon induction with doxycycline we saw interaction of PRUNE with microtubules (Fig. 3A), which was not observed in the empty vector clone (kinesin V, a microtubules-associated protein, used as control; Fig. 3A). Next, co-immunoprecitation assays were performed in which endogenous tubulins, in particular β-and α-tubulin, were readily able to immunoprecipitate Flag-tagged full-length PRUNE^FLAG^ (Fig. 3A). This interaction was found to be conserved with both PRUNE incorporating p.D30N and p.R297W mutations (PRUNE^D30N-FLAG^, PRUNE^R297W-FLAG^), although PRUNE^R297W-FLAG^ displayed less efficient binding (Supplementary Fig. 3A and B). Endogenous wild-type PRUNE protein subcellular localization was further evaluated by immunofluorescence analyses in HeLa cells during cell division, demonstrating overlap between PRUNE and of β-tubulin on astral and interpolar microtubules (prometaphase, metaphases, anaphase, and cytokinesis; Supplementary Fig. 4), indicative of a potentially important role in cellular division processes. To further investigate this role, we next explored a role of PRUNE in microtubule polymerization assay using GTP as substrate. Polymerization assays demonstrated that wild-type PRUNE as expressed in *E. coli* significantly enhances microtubule polymerization in nucleation, growth and steady-state (phase I-II-III; Fig. 3C and Supplementary Fig. 3F), and revealed a delay in microtubule formation affecting mainly growth phase associated with mutant PRUNE p.D30N protein, while PRUNE p.R297W negatively influences the early growth rate of microtubule polymerization processes, revealing distinct functional outcomes of each PRUNE mutation (Fig. 3C). Further, in studies in SH-SY5Y inducible clones expressing either wild-type or mutant (p.Asp30Asn and p.Arg297Trp) protein, mutant PRUNE-expressing cells were found to contain shortened microtubules compared with wild-type PRUNE, with aster diameter size measuring below 5 µm (Fig. 3B and Supplementary Table 3).

## Discussion

A great deal remains to be learned about the precise molecular mechanisms orchestrating normal cortical development and brain growth. However, significant advancements in this field have been made through genetic studies of inherited forms of primary microcephaly and malformations of cortical development.

Previous investigations show that PRUNE exhibits both exopolyphosphatase and phosphodiesterase activity, which has been suggested to enhance cellular proliferation and motility of breast cancer cells in the presence of its protein partner nucleoside diphosphate kinase 1 (NM23-H1) (Bilitou *et al.*, 2012).

Similarly, a correlation between PRUNE and metastasis formation in liver, gastric and oesophageal cancer with poor prognosis has been suggested (Oue *et al.*, 2007; Noguchi *et al.*, 2009; Hashimoto *et al.*, 2016). In previous studies, we demonstrated that silencing of the human *PRUNE1* gene in lung and breast cancer inhibits metastasis formation and cellular migration. The effect of this silencing was shown to primarily be mediated through inhibition of β-catenin phosphorylation, which in turn has a negative effect on the WNT signalling cascade (Carotenuto *et al.*, 2014; Freeman *et al.*, 2015). The protein structure of human PRUNE is thought to be similar to that of yeast PPASE. Single orthophosphates (Pi) are recognized as an important cellular energy source linked to phospholipid signalling and are generated through PPASE PRUNE activity utilizing polyphosphate (poly-P) substrates. Alteration of PPASE activity has never been linked to cancer progression (Tammenkoski *et al.*, 2008).

Studies of murine Prune and its binding partner metastasis suppressor NM23-M1, have suggested that they are likely to have an important role during early embryonic stages and in neuronal tissues actively undergoing proliferation, migration and differentiation (Carotenuto *et al.*, 2006). Here we identify proliferation and migration inducing activities for PRUNE in neuronal SH-SY5Y cells, which are significantly diminished by sequence alterations affecting amino acids p.Asp30 and p.Arg297 located in the Asp-His-His (DHH1 and DHH2) domains, likely to be fundamental for enzymatic activity. Consistent with this we previously demonstrated that substitution of amino acid Arg28, which is co-located with Asp30 in the DHH1 domain, significantly inhibits PRUNE enzymatic activity (Tammenkoski *et al.*, 2008). Asp30 and Arg297 are closely aligned (∼15 Å separation) alongside one another inside the predicted enzymatic pocket (Ahn *et al.*, 2001; Merckel *et al.*, 2001; Ugochukwu *et al.*, 2007; Carotenuto *et al.*, 2013) (Fig. 1E). Therefore p.Asp30Asn seems likely to modify DHH1 tertiary structure, while p.Arg297Trp may enhance DHH1-DHH2 domain interaction, and both alterations are likely to interfere with PRUNE functionality by augmenting catalytic pocket substrate binding and/or enzymatic activity (see Kcat/Km values in Supplementary Table 2). Given these findings, other factors such as PRUNE-interacting proteins (for example NME-NDPK or other partners) (Galasso and Zollo, 2009) may also regulate the rate of microtubule polymerization, with PRUNE acting as an enhancer of this activity.

The identification of *PRUNE1* mutations in the sizeable and diverse patient cohorts described here, together with confirmatory functional assays demonstrating a deleterious effect of gene mutation on PRUNE function, have enabled us to determine the core clinical phenotype associated with Prune syndrome and implicate altered tubulin dynamics as a credible mechanism by which loss of PRUNE function may result in the microcephaly, additional cortical and subcortical abnormalities and the neurological phenotype observed.

Previous studies have shown that primary microcephaly may result from mutations in genes that disrupt neurogenesis by a number of mechanisms. However, the protein products of most of these genes have related functions and play a crucial role in the formation and behaviour of the microtubule cytoskeleton and mitotic phase of the cell cycle, including transcription regulation, cell cycle progression, and centrosome maturation (MCPH1, CENPJ, CDK5RAP2); dynein binding and centrosome duplication (NDE1); neuronal progenitor proliferation (ASPM and STIL) and mitotic spindle formation (WDR62). (Woods *et al.*, 2005; Francis *et al.*, 2006; Barkovich *et al.*, 2012). These fundamental cellular processes are of particular importance during the development of the human cortex, where disruption results in abnormalities of neuronal proliferation and migration.

Elucidation of the molecular causes of primary microcephaly has also revealed a wide spectrum of phenotypes associated with the causative genes. Mutation of *WDR62*, for example, also results in a variety of structural brain disorders including lissencephaly, cerebellar hypoplasia and hypoplasia of the corpus callosum (Yu *et al.*, 2010). Interestingly, two genes associated with primary microcephaly, *CEP152* (Guernsey *et al.*, 2010; Kalay *et al.*, 2011) and *CENPJ* (Bond *et al.*, 2005) have also been associated with the microcephalic primordial dwarfism family of disorders, a group of conditions with global growth failure. Other genes associated with microcephalic primordial dwarfism shown to cause defects in centrosomal and spindle microtubule function include: *PCNT, CENPE* and *POC1A* (Griffith *et al.*, 2008; Shaheen *et al.*, 2012; Mirzaa *et al.*, 2014). Notably, mutations in tubulin genes encoding different α- and β-tubulin isotypes (*TUBA1A*, *TUBA8* and *TUBB2B*, *TUBB3*, *TUBB5* and *TUBG1*), have previously been shown to be involved in a large spectrum of developmental brain disorders collectively referred to as tubulinopathies (Keays *et al.*, 2007; Abdollahi *et al.*, 2009; Jaglin *et al.*, 2009; Tischfield *et al.*, 2010; Breuss *et al.*, 2012; Poirier *et al.*, 2013; Bahi-Buisson *et al.*, 2014), further highlighting the critical role of the microtubule cytoskeleton in normal human nervous system development (Jaglin and Chelly, 2009; Tian *et al.*, 2010). The mutant proteins underlying these tubulinopathy disorders result in abnormal microtubule formation that affect multiple aspects of brain development mediated through impaired mitosis, neuronal migration and axonal pathfinding. A comparison of MRI neuroimaging from patients with TUBA1A-, TUBB2B- and TUBB3-associated disease revealed some consistent phenotypical outcomes with a combination of microcephaly, ventriculomegaly, abnormal gyral and sulcal patterns (including microlissencephaly, lissencephaly and pachygyria), cerebellar vermis hypoplasia, small or absent corpus callosum and small pons being highly suggestive of an underlying tubulin mutation (Mutch *et al.*, 2016). In the present study we have identified an interaction between PRUNE and β-tubulin, and impairment of microtubule polymerization in the presence of mutant PRUNE. It is perhaps therefore unsurprising that the intracranial abnormalities associated with abnormal neurodevelopment seen in Prune syndrome patients show some overlap with those seen in association with mutant tubulin subtypes and are consistent with mitotic and axonal pathfinding abnormalities, most notably microcephaly, white matter changes, thin or hypoplastic corpus callosum, and cerebellar involvement.

More recently, homozygous mutations in *TBCD*, a gene encoding one of the five tubulin-specific chaperones that are required for α/β-tubulin *de novo* heterodimer formation, have been described in association with an infantile onset neurodegenerative disorder characterized by developmental regression, seizures, optic atrophy and secondary microcephaly, cortical atrophy with delayed myelination, cerebellar atrophy and thinned corpus callosum. Although patients with Prune syndrome manifest abnormal neurology from birth, the neurological impairment, microcephaly and cortical involvement appears to be progressive and there are some striking similarities in the neurodegenerative pattern and in white matter abnormalities seen on neuroimaging of these patients and that of patients harbouring recessive *TBCD* mutations and also some overlapping ophthalmological features such as the optic atrophy documented in the Italian family. Notably, reduced soluble α/β-tubulin levels and accelerated microtubule polymerization has been reported in fibroblasts derived from patients with biallelic *TBCD* mutations, while cellular proliferation was not markedly reduced. While more work is required to investigate this, the reduced rate of microtubule polymerization and cellular proliferation associated with the PRUNE mutations described here may in part provide an explanation for the more marked neurodevelopmental impairment seen in Prune syndrome patients (Edvardson *et al.*, 2016; Flex *et al.*, 2016; Miyake *et al.*, 2016; Pode-Shakked *et al.*, 2016).

Interestingly the clinical presentation in the Italian children, which included central hypotonia, profound global developmental delay, progressive microcephaly, cerebellar atrophy and epileptic encephalopathy with hypsarrhythmia and optic atrophy, fulfils the diagnostic criteria for PEHO (progressive encephalopathy with pedal oedema, hypsarrhythmia and optic atrophy) syndrome (Somer, 1993). Since the original clinical description of this condition in 1991, a number of different genes and modes of inheritance have been associated with clinical presentations said to be consistent with PEHO, or thought to be PEHO-like in nature, including *de novo* dominant variants in *CDKL5* and *KIF1A* and biallelic mutations in *CCDC8A* (Gawlinski *et al.*, 2016; Langlois *et al.*, 2016; Nahorski *et al.*, 2016)*.* The phenotype in the Italian family, as well as consistent overlapping clinical aspects present in the other families described here, provides cause to consider that *PRUNE1* should now also be added to the list of genes in which mutations may present in children with epileptic encephalopathy and PEHO-like features.

Given the similarity and overlapping nature of the phenotypes associated with mutation of tubulin isotypes and regulators, it has been proposed that they may function as part of the same subcellular protein complex or molecular pathway to regulate neuronal progenitor proliferation and migration where demand for tubulin is high. The overlapping clinical, molecular and genetic data presented here indicate that PRUNE may also play a role in this pathway, and lead us to suggest that Prune syndrome is a novel tubulinopathy disorder with both neurodevelopmental and degenerative components. This potential new role for PRUNE during microtubule polymerization and cytoskeletal rearrangements occurring during cellular division and proliferation therefore entails an important area for more in-depth future investigation.

## Web resources

The URLs for data presented herein are as follows:

GATK; http://www.broadinstitute.org/gatk/about/citing-gatk

VEP; http://www.ensembl.org/info/docs/variation/vep/index.html

SAMTOOLS; http://samtools.sourceforge.net

NHLBI Exome Sequencing Project (ESP); http://evs.gs.washington.edu/EVS/

Online Mendelian Inheritance in Man; http://www.ncbi.nlm.nih.gov/Omim

Pubmed; http://www.ncbi.nlm.nih.gov/pubmed/

Gene; http://www.ncbi.nlm.nih.gov/gene

ClustalW2; http://www.ebi.ac.uk/Tools/msa/clustalw2/

PolyPhen-2; http://genetics.bwh.harvard.edu/pph2/

SIFT; http://sift.jcvi.org/

PROVEAN; http://provean.jcvi.org/seqsubmit.php

GeneCards; http://www.genecards.org/

The 1000 Genomes Browser; http://browser.1000genomes.org/index.html

The Ensembl Project; http://www.ensembl.org/index.html

The National Center for Biotechnology; http://www.ncbi.nlm.nih.gov/

UCSC Human Genome Database; http://www.genome.ucsc.edu

## Supplementary Material

Supplementary Data
