# Supplementary material for: PRUNE is crucial for normal brain development and mutated in microcephaly with neurodevelopmental impairment
Source: Brain. 2017 Feb 28;140(4):940–52. doi: 10.1093/brain/awx014 (PMC5382943; doi:10.1093/brain/awx014)

## Slide 1
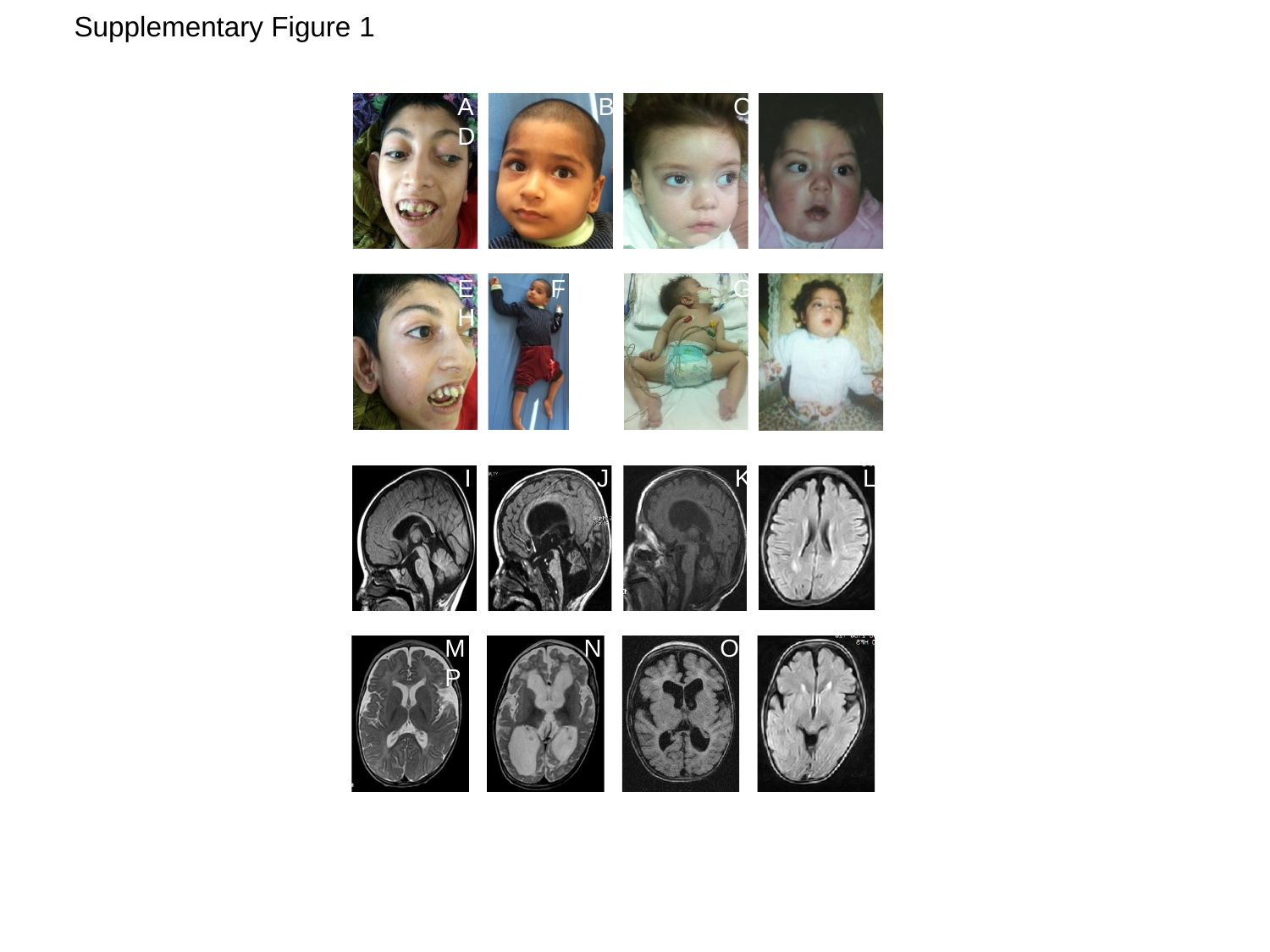

Supplementary Figure 1
A B C D
E F G H
 I J K L
M N O P

## Slide 2
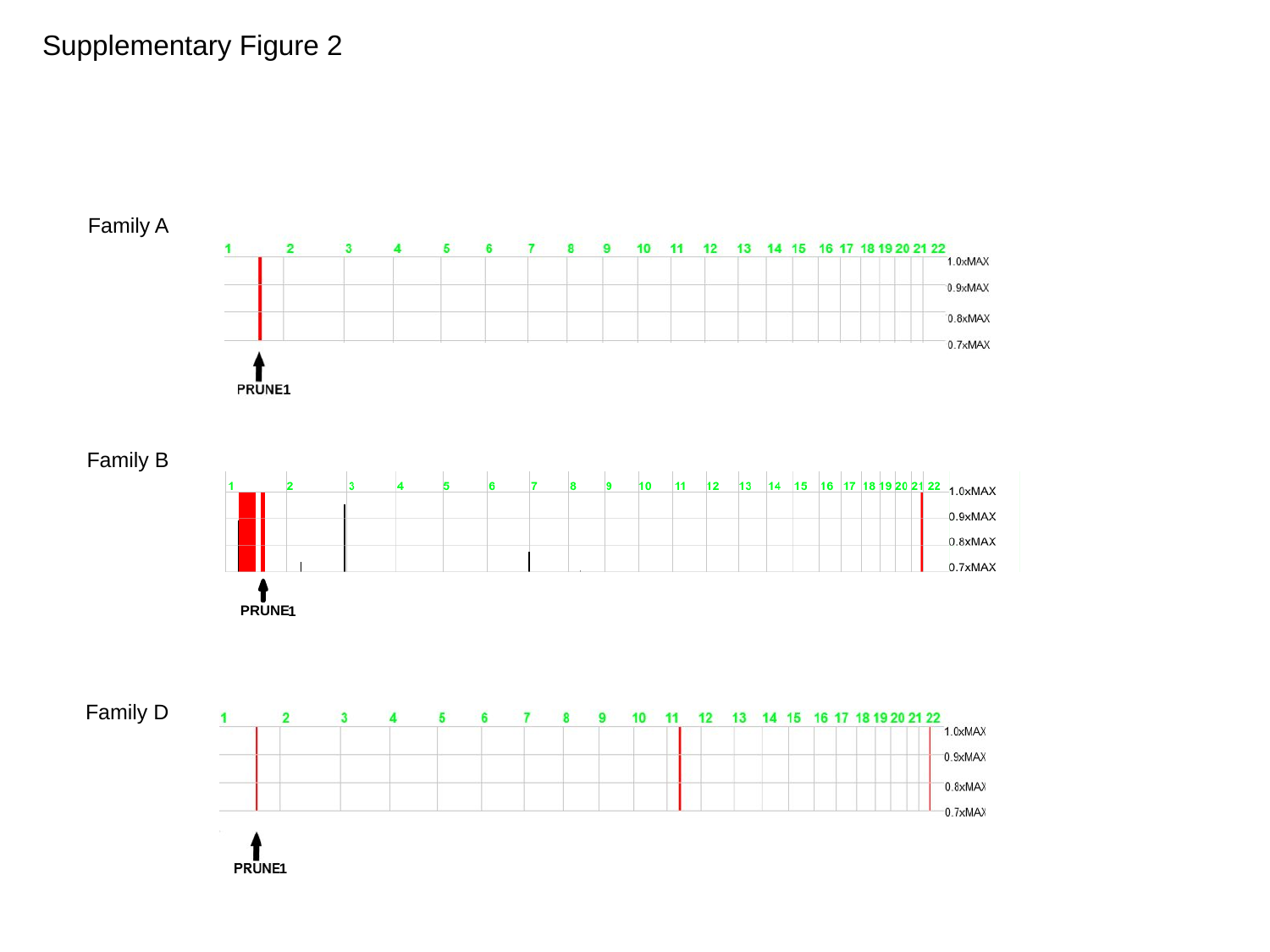

Supplementary Figure 2
Family A
Family B
PRUNE
Family D
1
1
1

## Slide 3
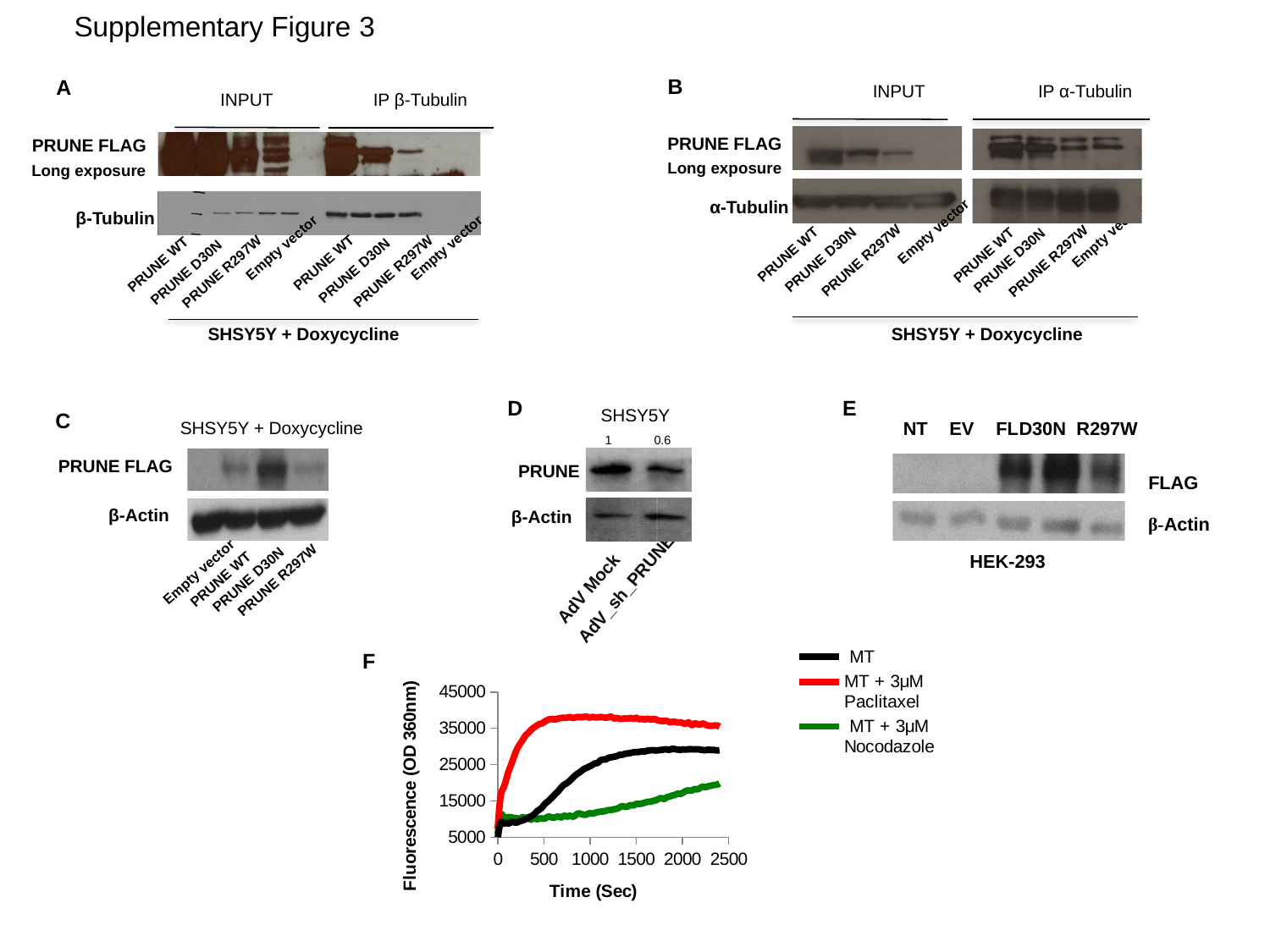

Supplementary Figure 3
B
INPUT
IP α-Tubulin
Prune FLAG
Long exposure
α-Tubulin
Empty vector
Prune WT
Prune D30N
Prune R297W
SHSY5Y + Doxycycline
A
INPUT
IP β-Tubulin
 Prune FLAG
Long exposure
β-Tubulin
Empty vector
Prune WT
Prune D30N
Prune R297W
Empty vector
Prune WT
Prune R297W
Prune D30N
SHSY5Y + Doxycycline
Empty vector
Prune WT
Prune D30N
Prune R297W
D
SHSY5Y
1
0.6
AdV_sh_Prune
AdV Mock
Prune
β-Actin
E
NT
EV
FL
D30N
R297W
FLAG
β-Actin
HEK-293
C
SHSY5Y + Doxycycline
Prune FLAG
β-Actin
Empty vector
PRUNE WT
PRUNE D30N
PRUNE R297W
### Chart
| Category | MT | MT + 3μM Paclitaxel | MT + 3μM Nocodazole |
|---|---|---|---|F

## Slide 4
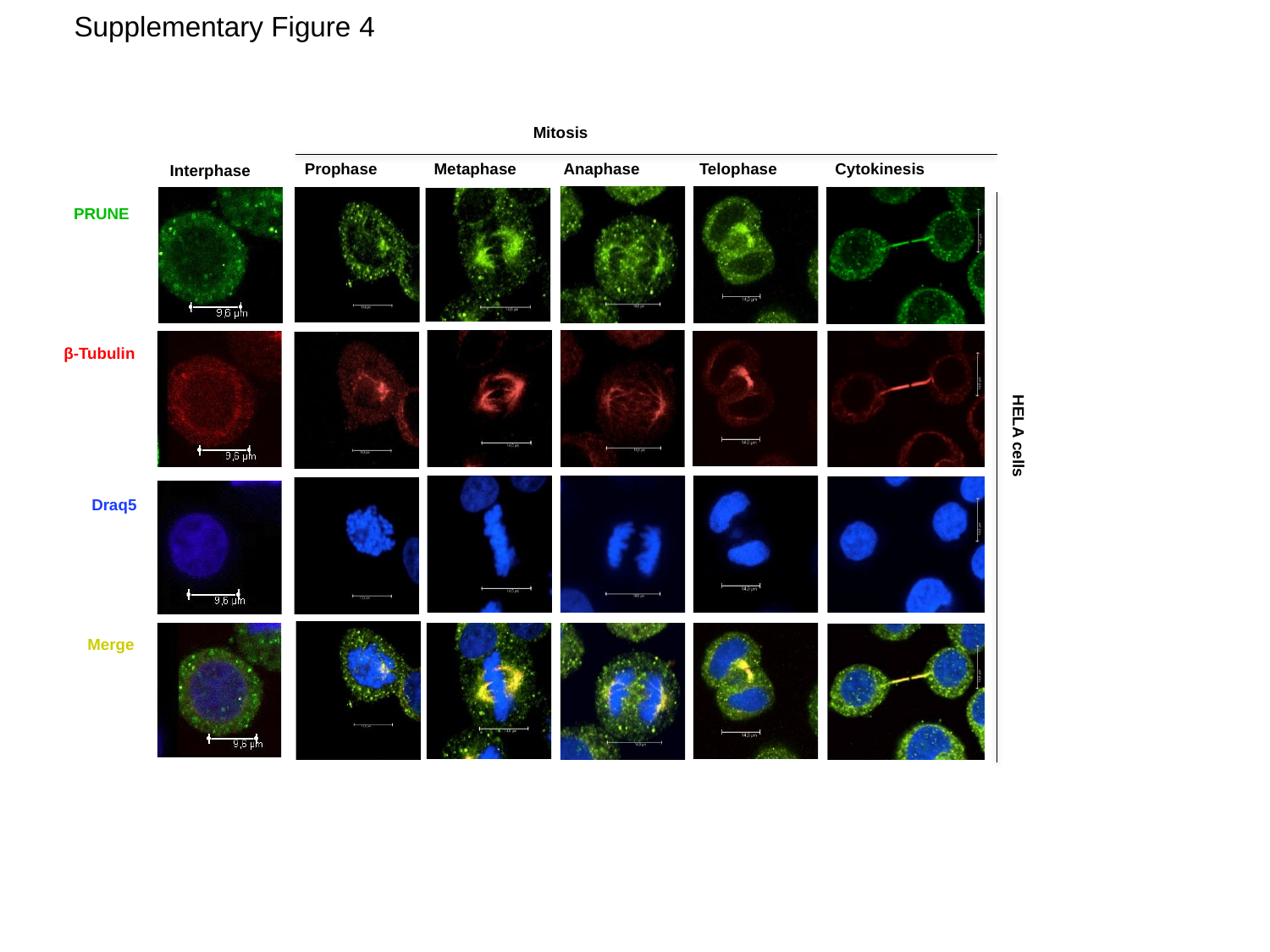

Supplementary Figure 4
Mitosis
Prophase
Metaphase
Anaphase
Telophase
Cytokinesis
Interphase
Prune
β-Tubulin
HELA cells
Draq5
Merge

## Slide 5
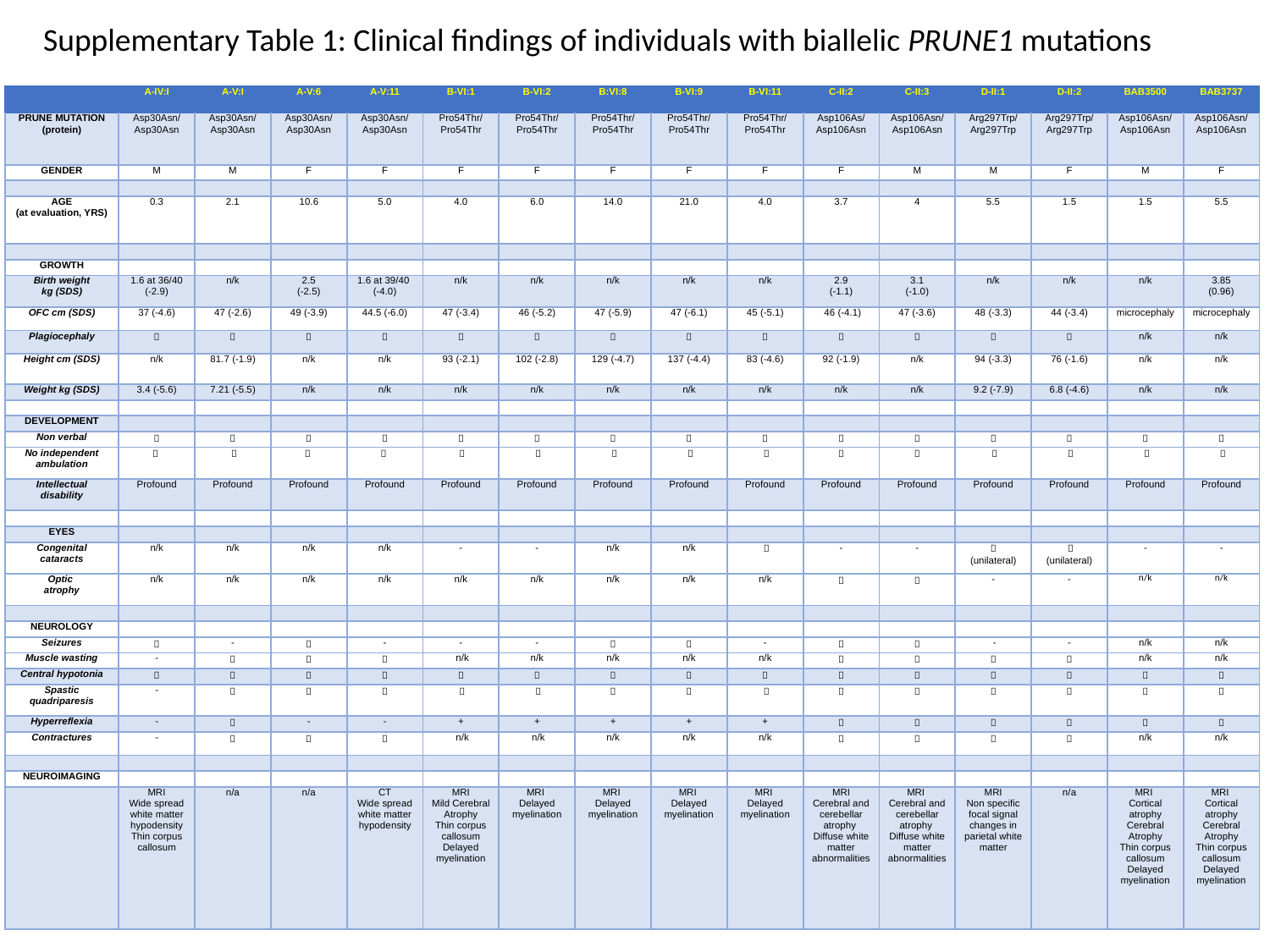

Supplementary Table 1: Clinical findings of individuals with biallelic PRUNE1 mutations
| | A-IV:I | A-V:I | A-V:6 | A-V:11 | B-VI:1 | B-VI:2 | B:VI:8 | B-VI:9 | B-VI:11 | C-II:2 | C-II:3 | D-II:1 | D-II:2 | BAB3500 | BAB3737 |
| --- | --- | --- | --- | --- | --- | --- | --- | --- | --- | --- | --- | --- | --- | --- | --- |
| PRUNE MUTATION (protein) | Asp30Asn/ Asp30Asn | Asp30Asn/ Asp30Asn | Asp30Asn/ Asp30Asn | Asp30Asn/ Asp30Asn | Pro54Thr/ Pro54Thr | Pro54Thr/ Pro54Thr | Pro54Thr/ Pro54Thr | Pro54Thr/ Pro54Thr | Pro54Thr/ Pro54Thr | Asp106As/ Asp106Asn | Asp106Asn/ Asp106Asn | Arg297Trp/ Arg297Trp | Arg297Trp/ Arg297Trp | Asp106Asn/ Asp106Asn | Asp106Asn/Asp106Asn |
| GENDER | M | M | F | F | F | F | F | F | F | F | M | M | F | M | F |
| | | | | | | | | | | | | | | | |
| AGE (at evaluation, YRS) | 0.3 | 2.1 | 10.6 | 5.0 | 4.0 | 6.0 | 14.0 | 21.0 | 4.0 | 3.7 | 4 | 5.5 | 1.5 | 1.5 | 5.5 |
| | | | | | | | | | | | | | | | |
| GROWTH | | | | | | | | | | | | | | | |
| Birth weight kg (SDS) | 1.6 at 36/40 (-2.9) | n/k | 2.5 (-2.5) | 1.6 at 39/40 (-4.0) | n/k | n/k | n/k | n/k | n/k | 2.9 (-1.1) | 3.1 (-1.0) | n/k | n/k | n/k | 3.85 (0.96) |
| OFC cm (SDS) | 37 (-4.6) | 47 (-2.6) | 49 (-3.9) | 44.5 (-6.0) | 47 (-3.4) | 46 (-5.2) | 47 (-5.9) | 47 (-6.1) | 45 (-5.1) | 46 (-4.1) | 47 (-3.6) | 48 (-3.3) | 44 (-3.4) | microcephaly | microcephaly |
| Plagiocephaly |  |  |  |  |  |  |  |  |  |  |  |  |  | n/k | n/k |
| Height cm (SDS) | n/k | 81.7 (-1.9) | n/k | n/k | 93 (-2.1) | 102 (-2.8) | 129 (-4.7) | 137 (-4.4) | 83 (-4.6) | 92 (-1.9) | n/k | 94 (-3.3) | 76 (-1.6) | n/k | n/k |
| Weight kg (SDS) | 3.4 (-5.6) | 7.21 (-5.5) | n/k | n/k | n/k | n/k | n/k | n/k | n/k | n/k | n/k | 9.2 (-7.9) | 6.8 (-4.6) | n/k | n/k |
| | | | | | | | | | | | | | | | |
| DEVELOPMENT | | | | | | | | | | | | | | | |
| Non verbal |  |  |  |  |  |  |  |  |  |  |  |  |  |  |  |
| No independent ambulation |  |  |  |  |  |  |  |  |  |  |  |  |  |  |  |
| Intellectual disability | Profound | Profound | Profound | Profound | Profound | Profound | Profound | Profound | Profound | Profound | Profound | Profound | Profound | Profound | Profound |
| | | | | | | | | | | | | | | | |
| EYES | | | | | | | | | | | | | | | |
| Congenital cataracts | n/k | n/k | n/k | n/k | - | - | n/k | n/k |  | - | - |  (unilateral) |  (unilateral) | - | - |
| Optic atrophy | n/k | n/k | n/k | n/k | n/k | n/k | n/k | n/k | n/k |  |  | - | - | n/k | n/k |
| | | | | | | | | | | | | | | | |
| NEUROLOGY | | | | | | | | | | | | | | | |
| Seizures |  | - |  | - | - | - |  |  | - |  |  | - | - | n/k | n/k |
| Muscle wasting | - |  |  |  | n/k | n/k | n/k | n/k | n/k |  |  |  |  | n/k | n/k |
| Central hypotonia |  |  |  |  |  |  |  |  |  |  |  |  |  |  |  |
| Spastic quadriparesis | - |  |  |  |  |  |  |  |  |  |  |  |  |  |  |
| Hyperreflexia | - |  | - | - | + | + | + | + | + |  |  |  |  |  |  |
| Contractures | - |  |  |  | n/k | n/k | n/k | n/k | n/k |  |  |  |  | n/k | n/k |
| | | | | | | | | | | | | | | | |
| NEUROIMAGING | | | | | | | | | | | | | | | |
| | MRI Wide spread white matter hypodensity Thin corpus callosum | n/a | n/a | CT Wide spread white matter hypodensity | MRI Mild Cerebral Atrophy Thin corpus callosum Delayed myelination | MRI Delayed myelination | MRI Delayed myelination | MRI Delayed myelination | MRI Delayed myelination | MRI Cerebral and cerebellar atrophy Diffuse white matter abnormalities | MRI Cerebral and cerebellar atrophy Diffuse white matter abnormalities | MRI Non specific focal signal changes in parietal white matter | n/a | MRI Cortical atrophy Cerebral Atrophy Thin corpus callosum Delayed myelination | MRI Cortical atrophy Cerebral Atrophy Thin corpus callosum Delayed myelination |

## Slide 6
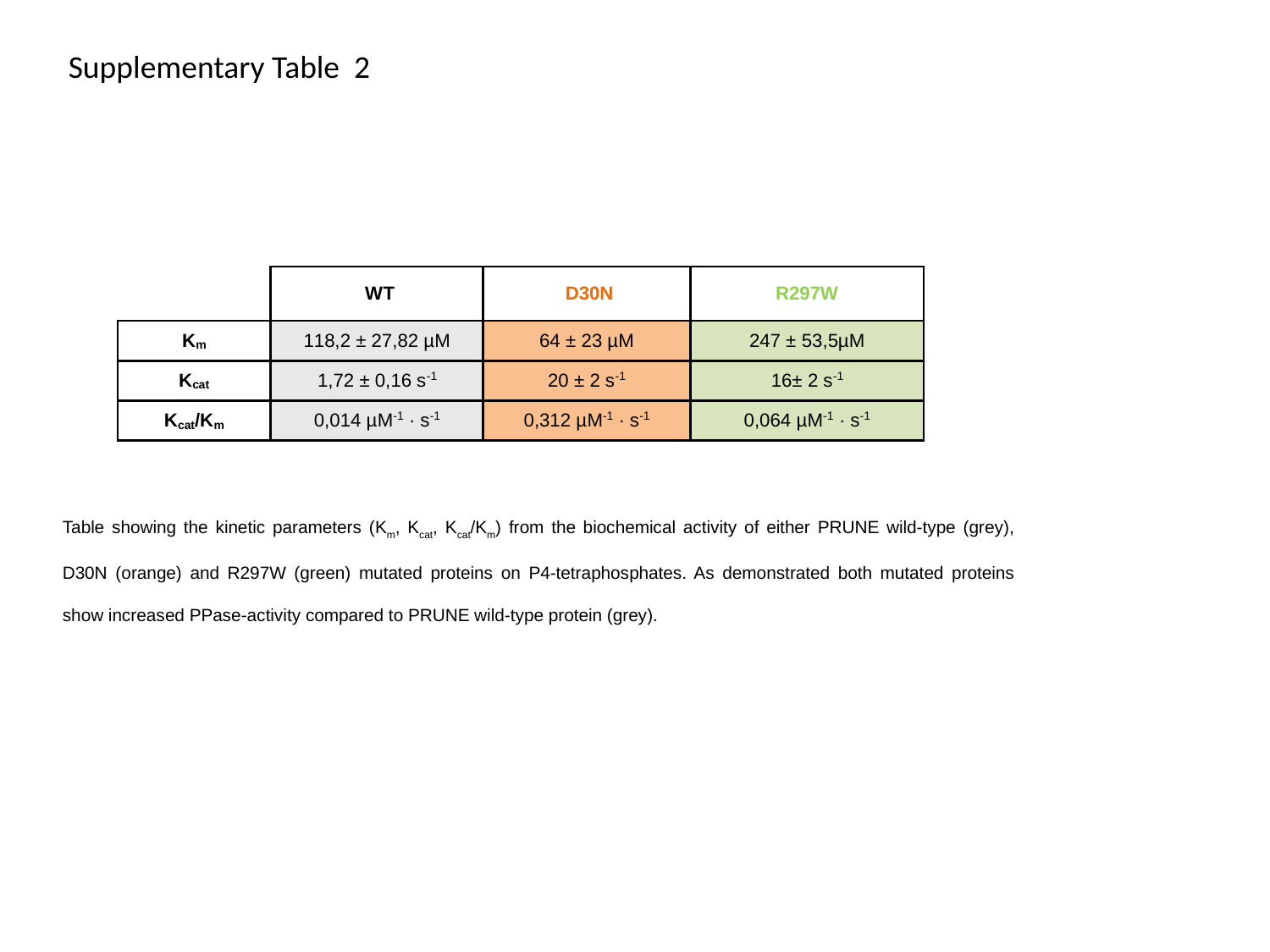

Supplementary Table 2
Table showing the kinetic parameters (Km, Kcat, Kcat/Km) from the biochemical activity of either Prune wild-type (grey), D30N (orange) and R297W (green) mutated proteins on P4-tetraphosphates. As demonstrated both mutated proteins show increased PPase-activity compared to Prune wild-type protein (grey).

## Slide 7
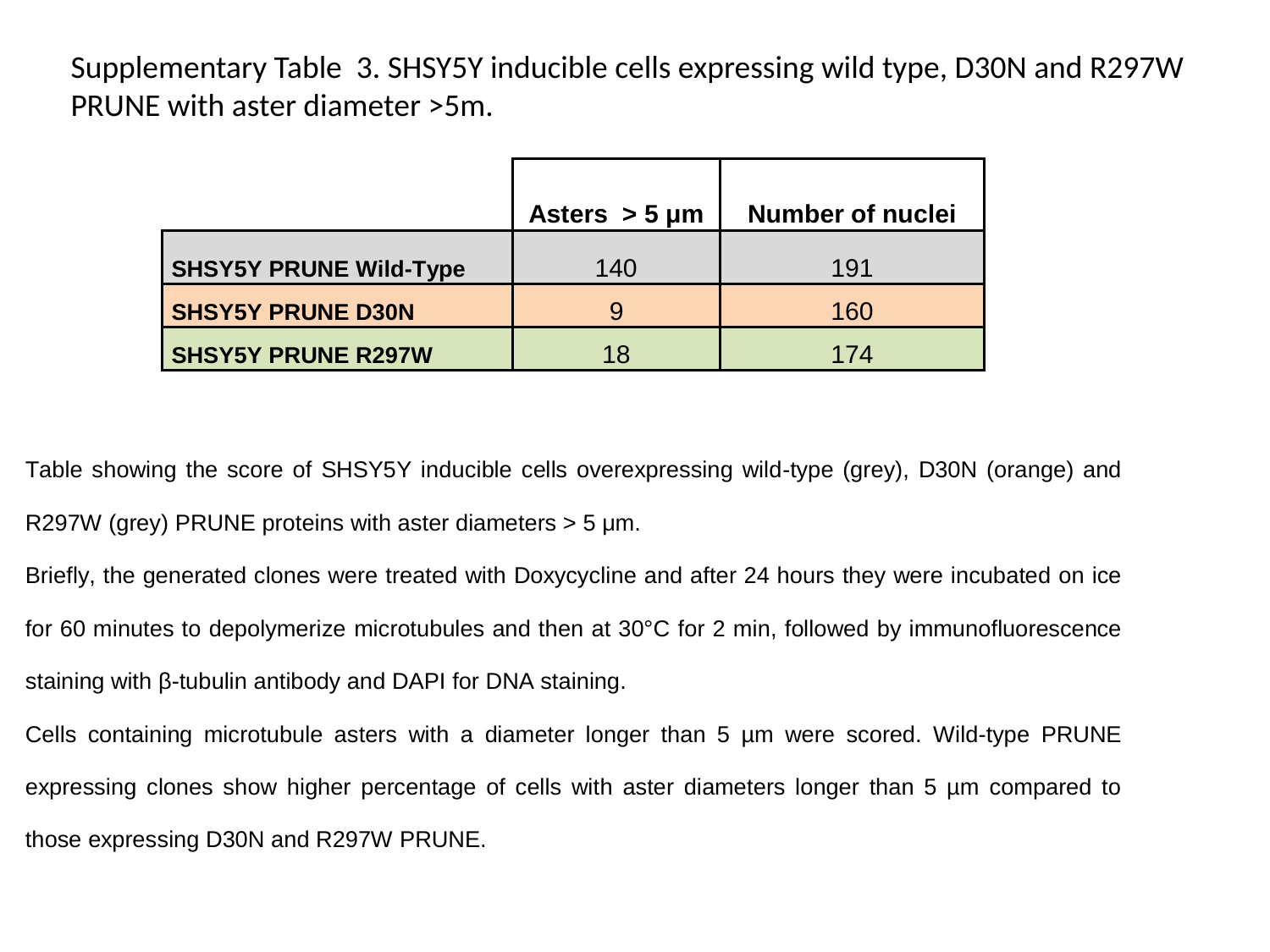

Supplement: Supplementary Data [file awx014_supp.zip › awx014-suppl_data/Supplementary Data 2017.pptx]
