# Supplementary material for: PRUNE is crucial for normal brain development and mutated in microcephaly with neurodevelopmental impairment
Source: Brain. 2017 Feb 28;140(4):940–52. doi: 10.1093/brain/awx014 (PMC5382943; doi:10.1093/brain/awx014)
Supplement: Supplementary Data [file awx014_supp.zip › awx014-suppl_data/Zollo et Supplementary Figure Legends 2017_FNL.docx]

**Supplementary material**

**PRUNE is crucial for normal brain development and mutated in microcephaly with neurodevelopmental impairment**

Massimo Zollo,^1,2,3*^ Mustafa Ahmed,^4,*^ Veronica Ferrucci,^1,2,3*^ Vincenzo Salpietro,^5*^ Fatemeh Asadzadeh,^1,2^ Marianeve Carotenuto,^1,2^ Reza Maroofian,^4^ Ahmed Al-Amri,^6,7^ Royana Singh,^8^ Iolanda Scognamiglio,^1,2^ Majid Mojarrad,^9,10^ Luca Musella,^1,2^ Angela Duilio,^11^ Angela Di Somma,^11^ Ender Karaca,^12^ Anna Rajab,^7^ Aisha Al-Khayat,^13^ Tribhuvan Mohan Mohapatra,^8^ Atieh Eslahi,^9^ Farah Ashrafzadeh,^9,14^ Lettie E. Rawlins,^4^ Rajniti Prasad,^15^ Rashmi Gupta,^8^ Preeti Kumari,^8^ Mona Srivastava,^8,16^ Flora Cozzolino,^2^ Sunil Kumar Rai,^8^ Maria Monti,^2,11^ Gaurav V. Harlalka,^4^ Michael A. Simpson,^17^ Philip Rich,^18^ Fatema Al-Salmi,^13^ Michael A. Patton,^4,13,19^ Barry A. Chioza,^4^ Stephanie Efthymiou,^5^ Francesca Granata,^20^ Gabriella Di Rosa,^21^ Sarah Wiethoff,^5^ Eugenia Borgione,^22^ Carmela Scuderi,^22^ Kshitij Mankad,^23^ Michael G. Hanna,^5,24^ Piero Pucci,^2,11^ Henry Houlden,^5^ James R. Lupski,^12,25,26,27^ Andrew H. Crosby^4^ and Emma L. Baple^4^

1 Dipartimento di Medicina Molecolare e Biotecnologie Mediche DMMBM, Università di Napoli Federico II, Via Sergio Pansini 5, Naples, 80131, Italy

2 CEINGE Biotecnologie Avanzate, Via Gaetano Salvatore 486, Naples, Italy

3 European School of Molecular Medicine, SEMM, University of Milan, Italy

4 Medical Research (Level 4), RILD Wellcome Wolfson Centre, University of Exeter Medical School, Royal Devon & Exeter NHS Foundation Trust, Barrack Road, Exeter, EX2 5DW, UK

5 Department of Molecular Neuroscience, UCL Institute of Neurology, London, UK

6 Section of Ophthalmology and Neuroscience, Leeds Institute of Biomedical and Clinical Sciences, University of Leeds, United Kingdom

7 National Genetic Centre, Directorate General of Royal Hospital, Ministry of Health, Muscat, Sultanate of Oman

8 Molecular Genetics, Department of Anatomy, Institute of Medical Sciences, Banaras Hindu University, Varanasi -221005, UP, India

9 Department of Medical Genetics, School of Medicine, Mashhad University of Medical Sciences, Mashhad, Iran

10 Medical Genetics Research Center, School of Medicine, Mashhad University of Medical Sciences, Mashhad, Iran

11 Dipartimento di Scienze Chimiche, Università Federico II, Naples, Italy

12 Department of Molecular and Human Genetics, Baylor College of Medicine, Houston, TX 77030, USA

13 Department of Biology, Sultan Qaboos University, PO Box 36, Post code 123, Sultanate of Oman

14 Department of Pediatric Neurology, Ghaem Medical Center, School of Medicine, Mashhad University of Medical Sciences, Mashhad, Zip Code- 9919991766, Iran

15 Department of Pediatrics, Institute of Medical Sciences, Banaras Hindu University, Varanasi -221005, UP, India

16 Department of Psychiatry, Institute of Medical Sciences, Banaras Hindu University, Varanasi -221005, UP, India

17 Department of Medical and Molecular Genetics, Division of Genetics and Molecular Medicine, King's College London, London, UK

18 Department of Neuroradiology, St. George’s Hospital, London, UK

19 Genetics Research Centre, St. George’s, University of London, London, SW17 0RE, UK

20 Unit of Neuroradiology, Department of Biomedical Science and Morphological and Functional Images, University of Messina, Messina, Italy

21 Unit of Child Neurology and Psychiatry, Department of Human Pathology of the Adult and Developmental Age, University of Messina, Messina, Italy

22 Unit of Neuromuscular disorders, IRCCS Oasi Maria SS Troina, Enna, Italy

23 Department of Neuroradiology, Great Ormond Street Hospital for Children, London WC1N 3JH, UK

24 MRC Centre for Neuromuscular Diseases, UCL Institute of Neurology, London WC1N 3BG, UK

25 Human Genome Sequencing Center, Baylor College of Medicine, Houston, TX 77030, USA

26 Department of Pediatrics, Baylor College of Medicine, Houston, TX 77030, USA

27 Texas Children's Hospital, Houston, TX 77030, USA

^*^These authors contributed equally to this work.

Correspondence may be addressed to: Dr Emma L. Baple,

Medical Research (Level 4),

RILD Wellcome Wolfson Centre,

Royal Devon & Exeter NHS Foundation Trust,

Barrack Road, Exeter, EX2 5DW, UK.

E-mail: [E.Baple@exeter.ac.uk](mailto:E.Baple@exeter.ac.uk)

Correspondence may also be addressed to: Professor Massimo Zollo (Functional studies)

Dipartimento di Medicina Molecolare e Biotecnologie Mediche,

Università degli Studi di Napoli Federico II,

Via Sergio Pansini 5, 80131 Naples, Italy

Email: massimo.zollo@unina.it

Correspondence may also be addressed to: Professor Andrew H. Crosby (Genetic studies)

Medical Research (Level 4),

RILD Wellcome Wolfson Centre,

Royal Devon & Exeter NHS Foundation Trust,

Barrack Road, Exeter, EX2 5DW, UK.

E-mail: A.H.Crosby@exeter.ac.uk

Correspondence may also be addressed to: Professor Henry Houlden (Italian family)

Department of Molecular Neuroscience

UCL Institute of Neurology

London WC1N 3BG, UK

Email: h.houlden@ucl.ac.uk

**Running title**

PRUNE is crucial for normal brain development

**Figure legends**

**Supplementary Figure 1: Clinical features of individuals with biallelic *PRUNE1* mutations.** (**A**-**E**) Facial appearance of patients with Prune syndrome. With increasing age, the sloping forehead and large prominent ears and eyes consistent with microcephaly become more apparent. (**F**-**H**) Central hypotonia in Prune syndrome patients. (**I**-**J**) Sagittal T1-weighted images of patient C-II:3 performed at 6 months of age (**I**) and 16 months of age (**J)** showing progressive global brain atrophy but more specifically evidence of cerebellar and brain stem atrophy, which is out of proportion to the cerebral atrophy. (**M**-**N**) Axial T2-weighted images performed at 6 months of age (**M**) and 16-months of age (**N**) in the same child (C-II:3), showing progressive diffuse white matter abnormalities along with progressive brain atrophy. **(K)** Axial inversion recovery and (**O**) sagittal T1-weighted MRI sequences performed in patient C-II:2 at 24 months of age shows generalized brain volume loss, but with specific evidence of cerebellar atrophy and a diffuse white matter signal abnormality as was seen in her sibling. (**L,P**) Axial images of patient D-II:1 showing plagiocephaly and non-specific focal signal changes in parietal white matter. There is mild widening of the Sylvian fissures, frontal sulci and frontal horns of the lateral ventricles suggesting some underdevelopment or atrophy of the frontal lobes. Cerebral white matter volume is otherwise preserved, no cortical malformation is noted and basal ganglia and thalami appear normal. The cerebellar sulci, fourth ventricle and post fossa CSF spaces are in general prominent although no cerebellar atrophy was noted (images not shown).

**Supplementary Figure 2: Homozygosity maps of affected family members, and disease gene identification**

**(Family A, B & D)** Genome-wide SNP microarray scan using Illumina Human CytoSNP-12 Beadchip arrays identified notable regions of homozygosity peculiar to affected individuals in each family, indicated by homozygosity plotting, of chromosome 1q21.2-1q23.1.

**Supplementary Figure 3**

**(A**-**B)** Protein extracts from SHSY5Y inducible cells (empty vector, PRUNE wild-type, D30N and R297W) were incubated with the anti-β-tubulin (**A**) or α-tubulin antibodies (**B**) to immunoprecipitate Flag-tagged PRUNE. Long exposures are shown indicating the presence of a band of the expected size (60 kDa) detected by western blotting using an anti-Flag antibody in the IP fraction from wild-type, D30N and R297W PRUNE-expressing clones indicating binding of PRUNE wild-type, D30N and R297W with both β- and α-tubulin. **(C)** SDS/PAGE followed by immunoblotting with an anti-FLAG antibody performed on whole protein extracts from SHSY5Y inducible clones upon treatment with doxycycline for 24 hours (β-Actin as loading control). **(D)** Immunoblotting showing prune expression in SHSY5Y cells treated with Ad-Mock and Ad-sh_prune (β-Actin as loading control). The relative densitometric analysis shows the downregulation (~40%) of PRUNE expression in sh-prune treated SHSY5Y cells (β-Actin as loading control). **(E)** Total protein from HEK293 after transfection subjected to immunoblotting with anti-Flag antibody to provide the expression control (β-Actin as loading control). **(F)** Graph showing the standard polymerization reaction alone and in the presence of 3μM paclitaxel (red) or 3 μM nocodazole (green) as respectively positive and negative control. Excitation was performed at 360 nm and emission at 420 nm with analyses performed using EnSpire manager software to evaluate the maximum absolute slope of each curve.

**Supplementary Figure 4**

Distribution of endogenous PRUNE (green), β-tubulin (red) and DNA (blue) in asynchronous HELA cells stained with antibodies against PRUNE, β-tubulin and Draq5 (for DNA staining). Co-localization of PRUNE and β-tubulin immunofluorescence is yellow in overlay. Scale bar equals 5µm. PRUNE colocalizes with β-tubulin in mitotic cells during all the sub-phases of mitosis (prophase, metaphase, anaphase, cytokinesis). In contrast, this colocalization is not observed in interphase cells (**left**).

**Table legends**

**Supplementary Table 1. Clinical findings of individuals with biallelic *PRUNE1* mutations**

Abbreviations; SDS, standard deviation scores; (🗸), indicates presence of a feature in an affected subject; (-), indicates absence of a feature in an affected subject); n/k, not known; n/a, not available.

Height, weight and OFC Z-scores were calculated using a Microsoft Excel add-in to access growth references based on the LMS method^1^ using a reference European population^2^. Individuals BAB3500 and BAB3737 are numbered as according to Karaca *et al*, 2015.

**Table references**

1.Pan H. CTJ. LMS growth, a Microsoft Excel add-in to access growth references based on the LMS method. Version 2.77.

Available from: <http://www.healthforallchildren.co.uk/>

2. Cole TJ, Freeman JV, Preece MA. British 1990 growth reference centiles for weight, height, body mass index and head circumference fitted by maximum penalized likelihood. *Stat Med* 1998; 17(4): 407-29.

**Supplementary Table 2. Kinetic parameters of wild-type, p.D30N and p.R297W PRUNE** showing the kinetic parameters (Km, Kcat, Kcat/Km) from the biochemical activity of either PRUNE wild-type (grey), D30N (orange) and R297W (green) mutated proteins on P4-tetraphosphates. Both mutated proteins show an increased PPAse-activity when compared to that of wild-type PRUNE protein (grey).

**Supplementary Table 3. SHSY5Y cell clones expressing wild-type, D30N and R297W PRUNE with aster diameter >5 μm**

Table showing the score of SHSY5Y inducible cells overexpressing wild-type (grey), D30N (orange) and R297W (grey) prune proteins with aster diameters >5 μm (clones treated with doxycycline followed by immunofluorescence staining with β-tubulin antibody and DAPI for DNA staining). Cells containing microtubule asters with a diameter longer than 5 µm were scored. Wild-type PRUNE expressing clones show a higher percentage of cells with aster diameters longer than 5 µm compared to those expressing p.D30N and p.R297W PRUNE.
